# Genetic screening of α-thalassemia fusion gene using routine flow-through hybridization

**DOI:** 10.3389/fgene.2024.1460974

**Published:** 2024-11-12

**Authors:** Yingbei Huang, Aiping Ju, Lihong Zheng, Biqiu Xu, Liang Liang, Youqiong Li

**Affiliations:** ^1^ Department of Obstetrics and Gynecology, Huadu maternal and Neonatal Healthcare Hospital of Guangzhou, Huzhong Hospital, Guangzhou, Guangdong, China; ^2^ Department of Medical Laboratory, Huadu maternal and Neonatal Healthcare Hospital of Guangzhou, Huzhong Hospital, Guangzhou, Guangdong, China; ^3^ Center for Medical Genetics and Prenatal Diagnosis, People’s Hospital of Guangxi Zhuang Autonomous Region, Nanning, Guangxi, China

**Keywords:** fusion gene, -α 4.2, thalassemia, Gap-PCR, flow-through hybridization

## Abstract

**Objective:**

The fusion gene is a rare form of α-thalassemia. Patients carrying the fusion gene could be misdiagnosed as normal or -α^4.2^deletion by the conventional thalassemia detection methods. The aim of this study was to present the detection of fusion genes using routine flow-through hybridization, as well as to analyze hematological and molecular characteristics.

**Methods:**

Samples were collected at our hospital from January 2019 to January 2024. Common thalassemia mutations in the Chinese population were conducted by flow-through hybridization. Samples showing faint coloration at the -α^4.2^ mutation site on hybridization membrane were considered suspicious. Samples detected as suspicious for -α^4.2^deletion were rechecked by conventional Gap-PCR. Those samples suspected of having -α^4.2^deletions were finally confirmed with specific primers for Gap-PCR and Sanger sequencing.

**Results:**

Of the 32,083 samples, 25 samples (0.08%) were detected as suspected of having -α^4.2^ deletion by flow-through hybridization. However, upon reevaluation wtih conventional Gap-PCR reagents capable of detecting -α^4.2^ deletion, all were found to be negative for the deletion. Specific primers for Gap-PCR were designed, and fusion gene fragments were amplified. DNA sequencing of the HBA gene showed a 7-base mutation corresponding to the α-thalassemia fusion gene. Among the 25 samples, 22 were heterozygous carriers. Three samples were combined: one with Hb QS, one with β-thalassemia, and one with Hb G-Honolulu.Most hematological indices and capillary electrophoresis results were in the normal reference range.

**Conclusion:**

The fusion gene was present in 0.08% of the population in the Guangzhou region of Guangdong province, southern China. Conventional genetic methods tend to misdiagnose the fusion gene but can be effectively screened with flow-through hybridization.

## Introduction

α-Thalassemia is a common monogenic disease characterized by decreased or absent synthesis of the α-globin gene cluster, leading to an imbalance in the ratio of α-/non α-globin chains (Baird et al., 2022; Lee et al., 2021). This cluster, which is part of chromosome 16p13.3’s telomeric region, consists of four functional genes and three pseudogenes (Higgs et al., 2012). Unequal crossover or gene conversion during genetic recombination is identified as a potential source of mutagenesis in human globin gene clusters (Borg et al., 2009).*HBA1* and *HBA2* are situated within two highly homologous segments spanning approximately 4kb, with their sequence similarity maintained through instances of gene conversion and uneven crossovers (Wu et al., 2015). This DNA region includes three closely homologous segments (X, Y, and Z) interspersed with nonhomologous regions (Hu and Shen, 1987). Additionally, due to significant sequence homology between the γ1-globin and α2-globin genes and between the ζ2-globin and ψζ1-globin genes, unequal homologous recombination commonly occurs among the two ζ- and two α-globin genes within this cluster (Law et al., 2006).

Recombination between two mismatched wild-type α gene clusters during meiosis can result in single α gene deletions, which are prevalent in α-thalassemia, such as -α^4.2^ and -α^3.7^ deletions (Embury et al., 1980). The X2 and X1 boxes, along with their normal homologous counterparts in the human α-globin gene cluster, have been investigated about the breakpoint region of the -α^4.2^ allele (Ou-Yang et al., 2004). Reciprocal recombination between Z boxes located 3.7 kb apart can result in a chromosome carrying only one functional α gene (-α^3.7^), causing α^+^-thalassemia, while a triplication allele (ααα^anti3.7^) does not typically cause thalassemic symptoms (Goossens et al., 1980). A rare form of α-thalassemia is caused by a fusion of the α2-globin gene with the Ψα1-globin gene sequence. Genetic analysis Kits for clinical use in China are available to detect common -α^3.7^ and -α^4.2^ deletions, but there are currently no primers designed to detect fusion genes (Hu et al., 2019; Li et al., 2019). This is because the population’s carrier rate of fusion genes is relatively low. Certain research has described that SEA deletion heterozygouscombined with fusion genes can lead to H disease (Huang et al., 2013). Thus, even though fusion genes are rare, screening and diagnosing them should be a concern for clinicians.

Due to the lack of extensive data on population-specific fusion genes, obtaining their hematological phenotype and molecular characteristics, as well as understanding whether they exacerbate clinical symptoms when combined with other types of thalassemia, is currently not feasible. In this study, we present a technique designed for routine screening of fusion genes and assessing the population carrier rate of such genes in the Guangdong province, Southern China.

## Materials and methods

### Subjects collection

A total of 32,083 samples for thalassemia molecular testing were randomly collected from January 2019 to January 2024 from the Huadu Maternal and Child Healthcare Hospital, Guangdong Province, China. Of these, 15,616 (48.7%) were male and 16,467 (51.3%) female. Inclusion criteria: 1. Results of complete blood cell count (CBC) and capillary electrophoresis (CE) were provided; 2. The patient (or the spouse) had a positive CBC or CE result; 3. Routine genetic analysis with flow-through hybridization was performed. Exclusion criteria: 1. Failure to provided results from CBC and CE; 2. Genetic analysis was not performed with flow-through hybridization. All participants in this study provided informed consent and signed. The study was approved by the Clinical Ethics Committee of the Huadu Maternal and Child Healthcare Hospital (2024-057).

### Hematological and hemoglobin (hb) analysis

Hematological parameters of these samples were obtained from an automatic analyzer (XN-1000; Sysmex Corporation, Kobe, Japan). The erythrocyte parameters we observed were Hb (reference: 115–165 g/L), mean corpuscular volume (MCV) (reference: 80-100 fL), and mean corpuscular Hb (MCH) (reference: 26–34 pg). Hb fractions were quantified using CE (CapillaryS2 Flex Piercing; Sebia, Lisses, Paris, France). The reference ranges for Hb A_2_ and Hb F are 2.4%–3.5% and 0%–5.0%, respectively. According to Guangzhou government policy, all couples of childbearing age are eligible for free genetic testing for thalassemia. A combined α-thalassemia and β-thalassemia genetic test is conducted when Hb A_2_>3.5%, and a α-thalassemia genetic test is performed when Hb A_2_<3.5%.

### Routine genetic analysis

**FIGURE 1:**
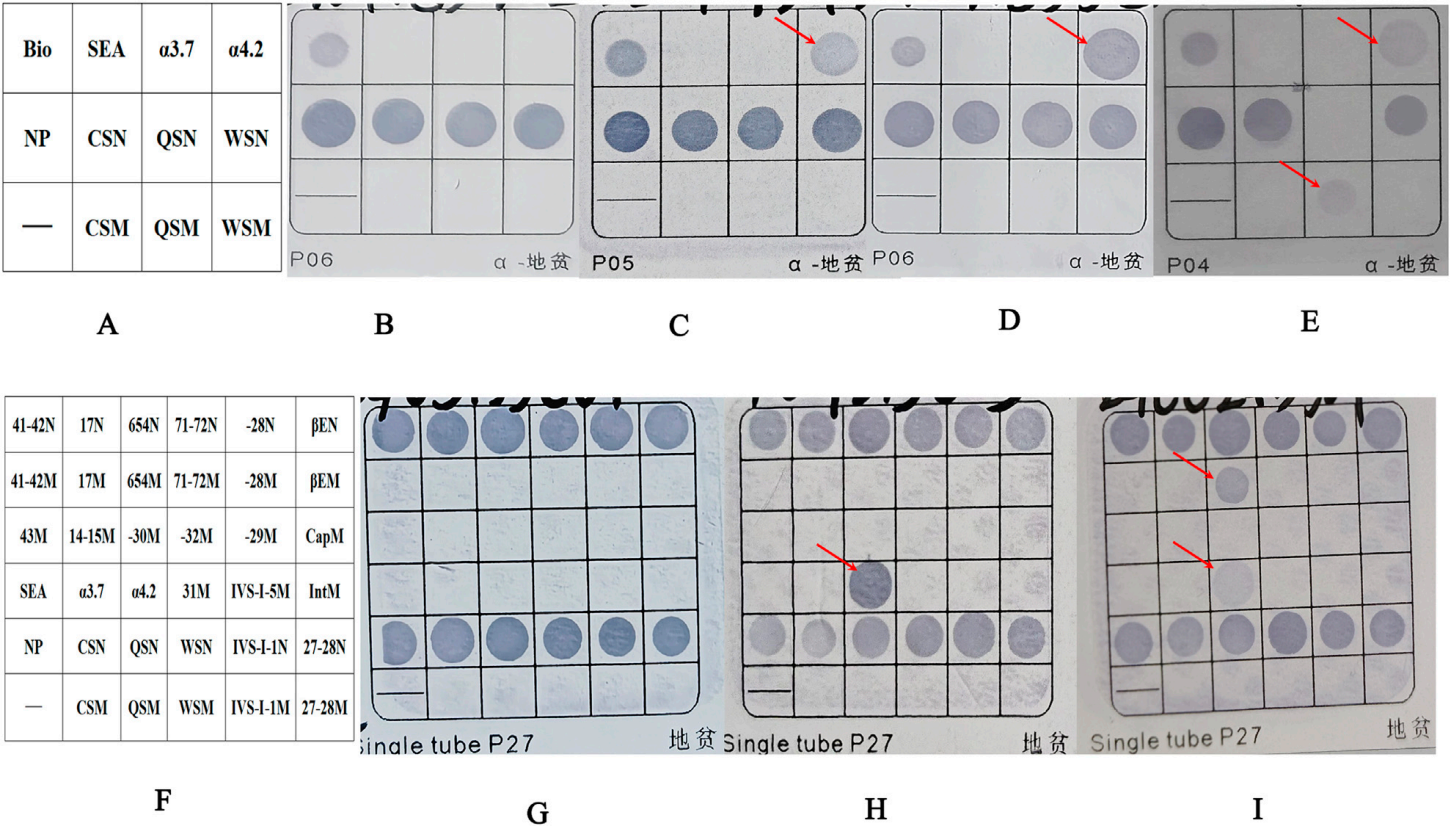
The results of the fusion genes were analyzed by flow-through hybridization. **(A)** Schematic representation of the α-thalassemia mutations on hybridized membrane strips. Bio as a sign that hybridization experiments were effective. **(B)** Normal individual. **(C)** Patient with fusion gene heterozygosity (faint coloration at the -α^4.2^ mutation site). **(D)** Patient with -α^4.2^deletionheterozygosity. **(E)** Patient with co-inherited fusion gene heterozygosity (faint coloration at the -α^4.2^ mutation site) and Hb QS heterozygosity. **(F)** Schematic representation of α-and β-thalassemia mutations on hybridized membrane strips. **(G)** Normal individual. **(H)** Patient with -α^4.2^deletionheterozygosity. **(I)** Patient with co-inherited fusion gene heterozygosity (faint coloration at the -α^4.2^ mutation site) and β^IVS−Ⅱ−654^heterozygosity. NP indicates a fragment for α2 gene, which served as a control for the heterozygous or homozygous status of--^SEA^, -α^4.2^, and–α^3.7^. The sites ending with “N” are considered wild-type loci, while those ending with “M” are mutation loci. For example, CSN represents a wild-type locus, and CSM represents a mutation locus. Abbreviations: QS, Hb Quong Sze (Hb QS); CS, Hb Constant Spring (Hb CS); WS, Hb Westmead (Hb WS).

Genomic DNA was extracted from peripheral blood with a DNA manual extraction kit (Chaozhou Hybribio Ltd., Chaozhou, China). Flow-through hybridization was used to detect the common α-thalassemia mutations in the Chinese population, including--^SEA^/, -α^3.7^/, -α^4.2^/, Hb Quong Sze (Hb QS), Hb Constant Spring (Hb CS), and Hb Westmead (Hb WS) (Chaozhou Hybribio Ltd., Chaozhou, China). Samples with Hb A_2_>3.5% were tested for 25 common mutations in α-thalassemia and β-thalassemia (Chaozhou Hybribio Ltd., Chaozhou, China). These mutations contained--^SEA^/, -α^3.7^/, -α^4.2^/, Hb QS, Hb CS, Hb WS, −32 (C>A), −30 (T>C), −29 (A>G), −28 (A>G), codons 14/15 (+G), codon 17 (A>T), codon 26 (G>A) (Hb E), codons 27/28 (+C), codon 31 (-C), IVS-Ⅰ-1 (G>T), IVS-Ⅰ-1 (G>A), IVS-Ⅰ-5 (G>C),codons 41/42 (-TCTT), codon 43 (G>T), codons 71/72 (+A), IVS-Ⅱ-654 (C>T), Cap+1 (A>C), Cap+1 (-AAAC), and initiation codon (ATG>AGG). Conventional Gap-PCR was used to detect the three common α-thalassemia deletions (--^SEA^/, -α^3.7^/, and -α^4.2^/) (Shenzhen Yaneng Biotechnology Company, Shenzhen, China). Samples showing faint coloration at the -α^4.2^ mutation site on hybridization membrane were considered suspicious (Figure 1). The samples suspected to be -α^4.2^ deletion would be rechecked using conventional Gap-PCR.

### Gap-PCR with specific primers

**FIGURE 2:**
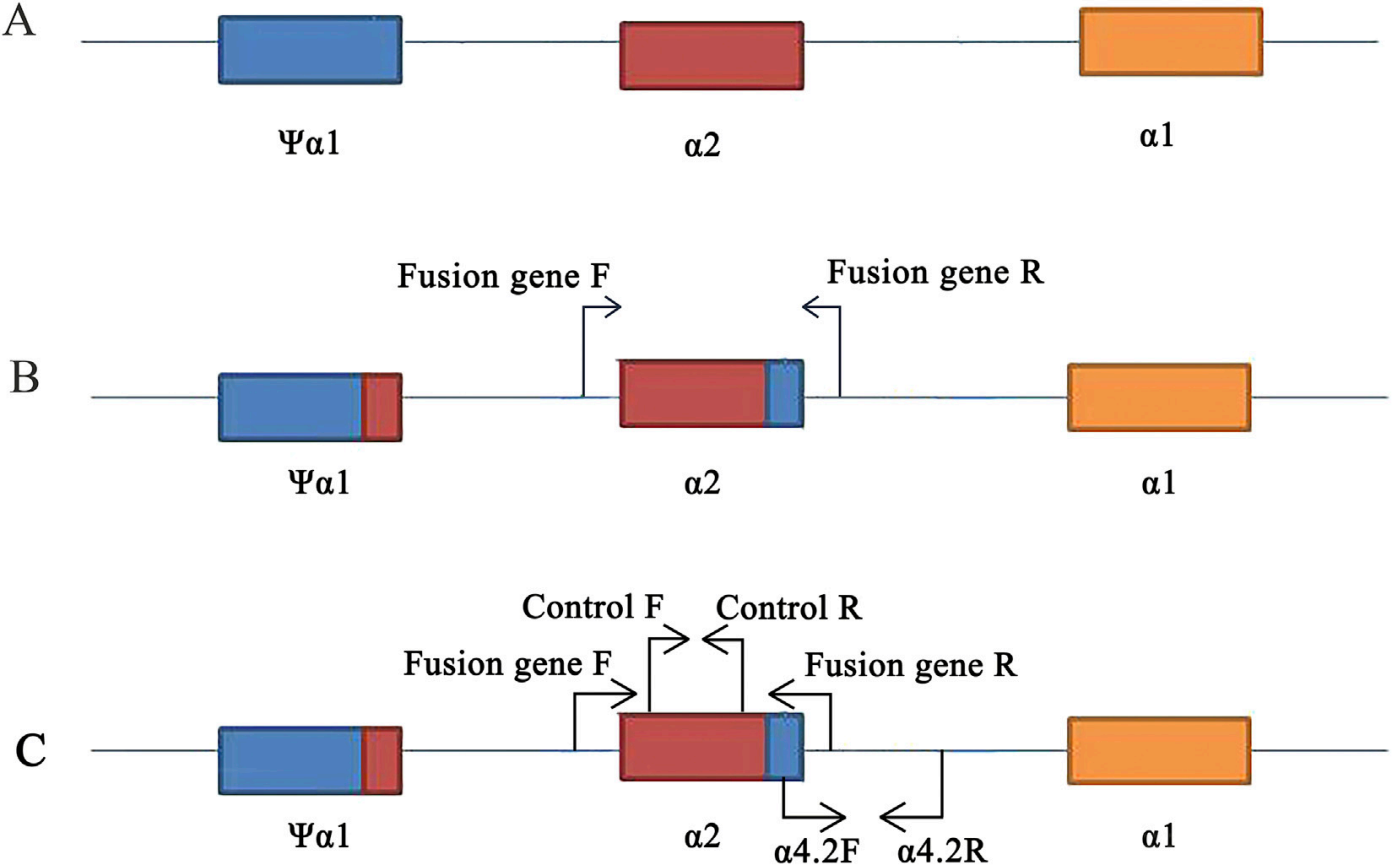
Primers design on the *HBA* gene. **(A)** Location of ψα1, α2, and α1 genes. **(B)** Design of specific primers for fusion gene. **(C)** Position of control and -α^4.2^deletion primers on the conventional reagent kit (flow-through hybridization).

Specific primers were designed for Gap-PCR to amplify the α-thalassemia fusion gene; the forward primer was 5′-GCG​AGC​GGG​ATG​GGC​GGG​AGT-3′, and the reverse primer was 5′-TGA​GTG​CTG​TGT​TGA​CCT​A-3' (Ju et al., 2023) (Figure 2B). A 50 μL reaction contained the following: 5 μL 10×PCR buffers, 5 μL Q-solution, 62.5 mmol/L MgCl_2_, 25 mmol/L dNTPs, 1.5 u Tap polymerase, 5 pmol/L forward primer, 5 pmol/L reverse primer, and 5 μL DNA template (QIAGEN, Hilden, Germany). PCR cycling conditions for optimal fragment amplification were 95°C for 15 min (1 cycle); 97°C for 50 s, 60°C for 60 s, and 72°C for 2 min (35 cycles); and 72°C for 10 min (1 cycle).

### Sanger sequencing

As in prevous literature (Li et al., 2018), primers were designed to amplify the *HBA1* and *HBA2* genes. The PCR products were sequenced on a 3500XL genetic analyzer (Applied Biosystems, Foster City, CA, United States).

## Results

### Genetic analysis for common thalassemia mutations

**FIGURE 3:**
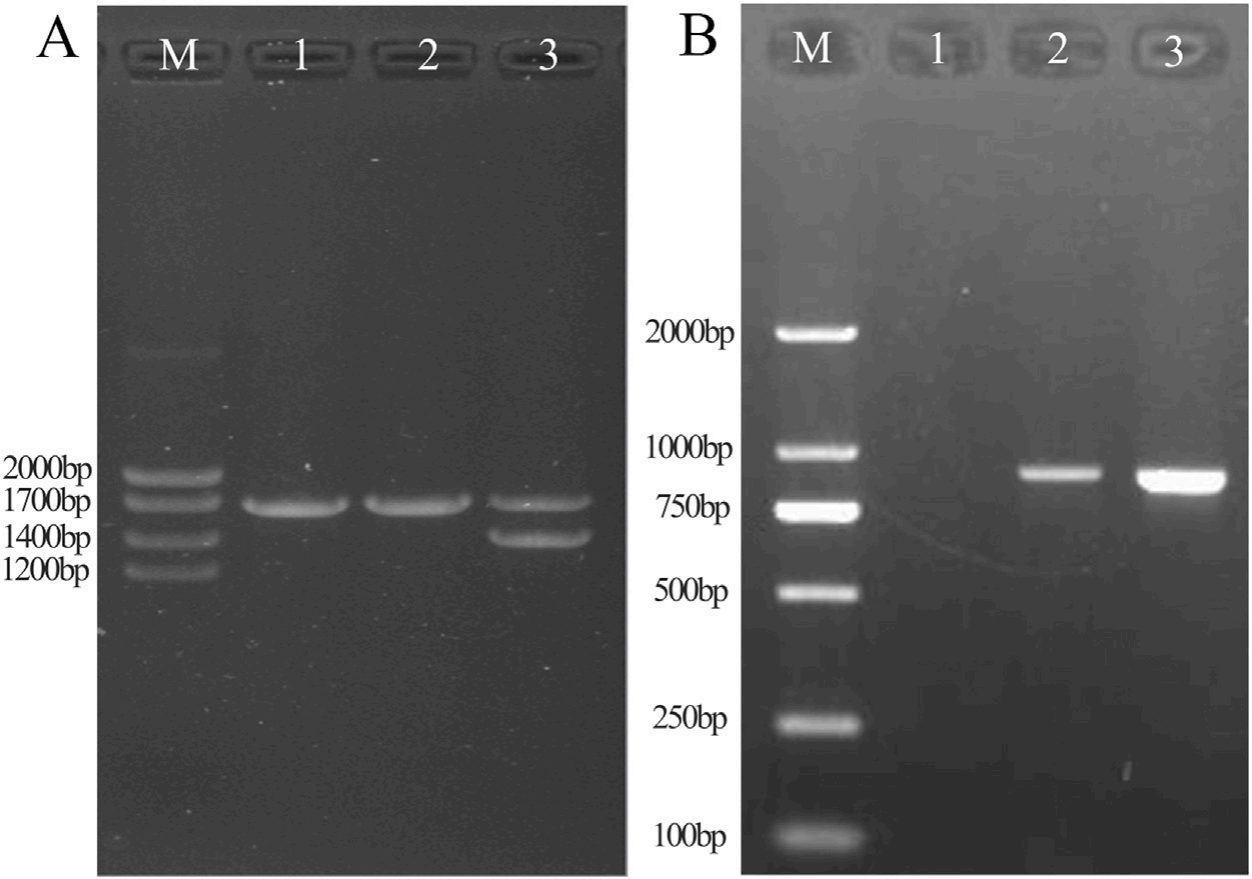
Results of conventional Gap-PCR **(A)** and specific primers for Gap-PCR **(B)**. A1: Normal individual. A2: Patient with fusion gene heterozygosity. A3: Patient with -α^4.2^ deletion heterozygosity. By comparison with the -α^4.2^ deletion positive control, it was shown that the sample was ruled out as an -α^4.2^ deletion. B1: Normal individual. B2: Patient with fusion gene heterozygosity. B3: Positive control for fusion gene. Gap-PCR with specific probes showed that the fusion gene samples amplified fragments consistent with the fusion gene positive control.

During January 2019 to January 2024, 25of the 32,083 samples were detected as suspected to be -α^4.2^ deletion with a detecting rate of 0.08% (25/32,083) by Flow-through hybridization. Those samples suspected of having a -α^4.2^ deletion had lighter coloration at the mutation site than the true -α^4.2^ deletion (Figure 1). Following two repetitions of the test, the -α^4.2^ mutation site exhibited no improvement in coloration. Finally, after rechecking with conventional Gap-PCR, they were ruled out as a -α^4.2^ deletion (Figure 3A). Among the 25 samples, 22 were heterozygous. Three samples were combined: one with Hb QS, one with β-thalassemia (β^IVS−Ⅱ−654^/), and one with unknown abnormal hemoglobin (Figure 1).

### Gap-PCR with specific primers

By Gap-PCR amplification, we obtained fragments ranging from Marker 750 bp to 1,000 bp, consistent with the positive sample for the fusion gene (Figure 3B). This finding indicated that the existence of a fusion gene rather than a -α^4.2^ deletion was responsible for the weak coloration of the -α^4.2^ deletion mutation site.

### Identification of fusion gene and abnormal hemoglobin by sanger sequencing

**FIGURE 4:**
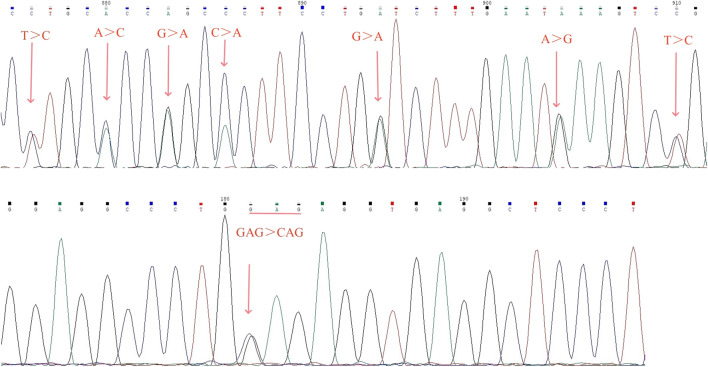
Results of fusion gene heterozygosity **(A)** and Hb G-Honolulu heterozygosity **(B)** by Sanger sequencing.

Sanger sequencing revealed there were seven conserved single bases (nt 876 C, nt 880 C, nt 883 A, nt 886 A, nt 894A, nt 904 G, and nt 910 C) in the PCR fragment, which were different from the corresponding nucleotide positions on the normal α2 gene (nt 34,528 T, nt 34,532 A, nt 34,535 G, nt 34,538 C, nt 34,546 G, nt 34556A, and nt 34,562 T; nucleotide positions from NG_000006) (Figure 4A). Sanger sequencing of the *HBA* gene identified the transversion mutation *HBA1*:c.91G>C in a sample with Hb variant, which resulted in an amino acid change from glutamic to glutamine at codon 30 of exon 1 in the heterzygous state, corresponding to Hb G-Honolulu (Figure 4B).

### Complete blood count test and Hb analysis

**FIGURE 5:**
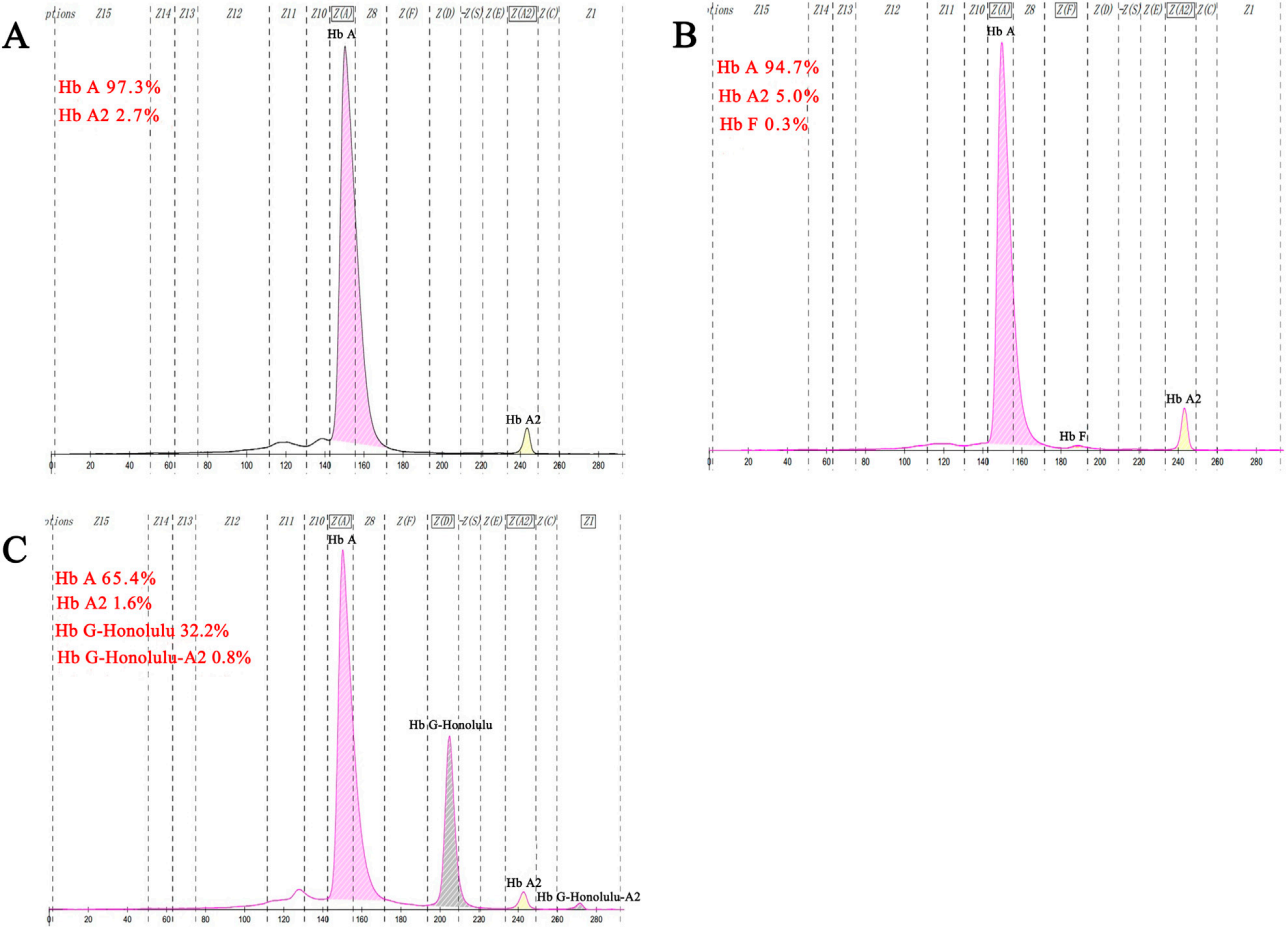
Results of fusion gene heterozygosity **(A)**, fusion gene heterozygositycombined with β^IVS−Ⅱ−654^heterozygosity **(B)**, fusion gene heterozygositycombined with Hb G-Honolulu heterozygosity **(C)** using CE.

Hematological analysis showed that Hb was below the reference value in four samples (16.0%, 4/25), MCV was below the reference value in 10 samples (40.0%, 10/25), and MCH was below the reference value in 10 samples (40.0%, 10/25).CE displayed three samples with Hb A_2_ below the reference value and one above the reference value (16.0%, 4/25). The CE of one sample suggested the presence of an Hb variant, located in the zone 6 (D zone) (Figure 5C). Generally, most of the hematological indices and CE results were in the normal reference range (Table 1).

**TABLE 1:** Hematologic phenotypes and genotypes of the fusion genes in this study.

Cases	Sex	Age(y)	Hb (g/L)	MCV (fL)	MCH (pg)	Hb A (%)	HbA_2_ (%)	Other Hb (%)	Genotype
1	F	28	126	78.7	25.5	65.4	1.6 + 0.8	Hb X 32.2	Fusion gene/α^CD30^α, β^N^/β^N^
2	M	26	156	82.9	26.1	97.0	3.0	0	Fusion gene/αα, β^N^/β^N^
3	M	31	157	78.5	26.1	97.5	2.5	0	Fusion gene/αα, β^N^/β
4	F	24	133	77.3	25.8	97.6	2.4	0	Fusion gene/αα, β^N^/β^N^
5	F	29	134	81.2	26.8	97.4	2.6	0	Fusion gene/αα, β^N^/β^N^
6	M	30	143	81.5	27	97.4	2.6	0	Fusion gene/αα, β^N^/β^N^
7	F	34	114	77.1	25.1	97.5	2.5	0	Fusion gene/αα, β^N^/β^N^
8	F	27	122	83.5	26.2	97.5	2.5	0	Fusion gene/αα, β^N^/β^N^
9	F	29	130	80.3	25.8	97.5	2.5	0	Fusion gene/αα, β^N^/β^N^
10	M	31	139	62.2	19.5	97.8	2.2	0	Fusion gene/α^QS^α, β^N^/β^N^
11	F	23	129	84.1	27.6	97.7	2.3	0	Fusion gene/αα, β^N^/β^N^
12	M	32	147	80.7	25.6	97.5	2.5	0	Fusion gene/αα, β^N^/β^N^
13	M	38	151	89.7	28.1	97.4	2.6	0	Fusion gene/αα, β^N^/β^N^
14	F	34	141	80.4	27.1	97.7	2.3	0	Fusion gene/αα, β^N^/β^N^
15	M	27	137	67.8	21.1	94.7	5.0	Hb F 0.3	Fusion gene/αα, β^IVS−Ⅱ−654^/β^N^
16	M	23	149	79.7	25.9	97.4	2.6	0	Fusion gene/αα, β^N^/β^N^
17	M	36	143	81.5	25.7	97.5	2.5	0	Fusion gene/αα, β^N^/β^N^
18	F	24	135	81.4	26.1	97.5	2.5	0	Fusion gene/αα, β^N^/β^N^
19	M	36	158	80.8	25.4	97.6	2.4	0	Fusion gene/αα, β^N^/β^N^
20	F	26	134	81.4	26.2	97.4	2.6	0	Fusion gene/αα, β^N^/β^N^
21	F	34	110	78.5	26.3	97.6	2.4	0	Fusion gene/αα, β^N^/β^N^
22	M	31	160	79.7	26.9	97.3	2.7	0	Fusion gene/αα, β^N^/β^N^
23	F	43	110	80.9	26.6	97.5	2.5	0	Fusion gene/αα, β^N^/β^N^
24	M	22	122	83.3	26.2	97.6	2.4	0	Fusion gene/αα, β^N^/β^N^
25	M	8 moth	107	70.8	22.8	-	-	-	Fusion gene/αα, β^N^/β^N^

## Discussion

Multigene families frequently experience homologous recombination. Unlike the common large fragment recombination genes (-α^3.7^/and -α^4.2^/), fusion genes resulting from mutations at multiple loci are less frequent, as reported in this study (Higgs, 2013). It involved a fusion between the α2 and the Ψα1 genes sequence, with mutations observed at seven sites (nt 34,528 C, nt 34,532 C, nt 34,535 A, nt 34,538 A, nt 34,546 A, nt 34556G, and nt 34,562 C) on the α2 gene. Our assay results were consistent with the study of Huang et al.and Liu et al., (2016), Huang et al. (2013). However, there is a discrepancy with the study by Hu et al., where they attributed the fusion gene to an 8-base mutation in the sequence (Hu et al., 2019). This discrepancy may be attributed to regional and ethnic differences; their study involved patients from the Li minority in Hainan province, whereas our study subjects were from the Han population in Guangdong province.

Fusion genes are predominantly observed in clinical cases of leukemia and cancer, with few reports of fusion genes associated with thalassemia (Gkountakos et al., 2024; Fouad and Eid, 2023). This study represents the first report on the prevalence of fusion gene carriers in the Chinese population, estimated at 0.08% (25 out of 32,083 individuals). Interestingly, all identified fusion gene carriers were from the Guangzhou region of Guangdong Province, with no detections in other examined populations. This was the same as the population carrier rate (0.08%) of the fusion gene that we previously reported in the Huadu district (Ju et al., 2023). Huadu District is part of the Guangzhou region and is located in the northern part of the Guangzhou region.This suggests that Guangzhou has a higher prevalence of fusion gene carriers in China.

Due to the relatively low carrier rate of fusion genes in the population and the focus of Chinese laboratory test kits on common mutations, conventional molecular tests often fail to detect fusion genes. In our laboratory, we utilize test kits with flow-through hybridization technology. Through our experience, we occasionally observed faint coloration at the -α^4.2^ deletion site. Using Gap-PCR with specific primers, we obtained an amplified fragment of the fusion gene (length was 901 bp). Subsequently, Sanger sequencing confirmed a mutation involving seven bases due to the fusion of the α2 segment of α-globin genes with the Ψα1 segment sequence. This mutation positioned the sequence above the α-thalassemia deletion amplification system within the normal range of internal reference (control) sequences (Figure 2C). This suggested that fixed hybridization probes with -α^4.2^ deletion located on the membrane have low quantitative complementarity to DNA carrying fusion genes. It caused non-specific hybridization with the -α^4.2^ deletion probe, resulting in weak coloration at the -α^4.2^ deletion site and potentially leading to misdiagnosis of a -α^4.2^deletion heterozygote. In our study, all 25 cases showing weak coloration at the -α^4.2^deletion site were confirmed to be cases of fusion genes. This indicated the utility of flow-through hybridization kits as a screening method for detecting fusion genes in regions with high population carriers of fusion genes.

This phenomenon was also observed in the PCR-RDB methodology. Liu et al. reported the absence of coloration on membrane strips with the normal reference probe in -α^3.7^ deletion heterozygotes affected by fusion genes (Liu et al., 2016). This occurs because the fusion gene disrupts the binding ability of the probe to the normal control sequence, resulting in a lack of color development. However, the lack of coloration on these RDB membrane strips is not specific. In cases where α^0^-thalassemia is combined with α^+^-thalassemia or α^0^-thalassemia with another α^0^-thalassemia, the control site of the normal reference sequence on the membrane strip also fails to show color. Therefore, this method cannot effectively screen for fusion genes as the flow-through hybridization kit does.

This fusion gene changed the 3′UTR of the α2 gene and caused a mutation in the polyadenylation signal, which produces an extensive transcript of the α2 gene associated with α^+^-thalassemia. As shown in Table 1, most hematological indices remained unchanged in heterozygous carriers of the fusion gene. However, individuals co-inheriting the fusion gene and α^0^-thalassemia can manifest as Hb H disease (Huang et al., 2013). Using complete blood count, approximately 60.0% (15/25) of patients might go undetected. In contrast, hemoglobin analysis could miss 80.0% (20/25) of cases. In China, conventional used assay kits include Gap-PCR, PCR-RDB, high-resolution melting and flow-through hybridization. To date, no fusion genes have been reported by the three methods other than flow-through hybridization. Therefore, detection of the fusion gene is challenging with commonly used test kits outside of flow-through hybridization in China. Accurate diagnosis of fusion genes is crucial for offering genetic counseling and advancing hematology practices, particularly in regions of China where fusion genes are common. In areas where fusion genes are not prevalent, there may be false positives when using flow-through hybridization because the faint staining is caused by other mutations.

## Data Availability

The data presented in this study can be found in online repositories. The names of the repository/repositories and accession number(s) can be found in the article/supplementary material.
